# Mechanical Thrombectomy via Transradial Approach for Posterior Circulation Stroke: A Systematic Review and Meta-Analysis

**DOI:** 10.7759/cureus.26589

**Published:** 2022-07-05

**Authors:** Hassan Kobeissi, Sherief Ghozy, Michael Liu, Gautam Adusumilli, Cem Bilgin, Ramanathan Kadirvel, David F Kallmes, Waleed Brinjikji

**Affiliations:** 1 Medicine, Central Michigan University College of Medicine, Mt. Pleasant, USA; 2 Neuroradiology, Mayo Clinic, Rochester, USA; 3 Neurology, Mayo Clinic, Rochester, USA; 4 Radiology and Neurosurgery, Stanford University, Stanford, USA; 5 Radiology, Mayo Clinic, Rochester, USA

**Keywords:** endovascular, ischemic, stroke, transradial, mechanical thrombectomy (mt)

## Abstract

Mechanical thrombectomy for acute ischemic stroke (AIS) is traditionally performed via transfemoral access. While the majority of AISs are due to anterior circulation large vessel occlusions (AC-LVO), we performed a systematic review and meta-analysis to examine the feasibility of and outcomes following a transradial artery access for posterior circulation large vessel occlusion (PC-LVO) strokes.

A systematic literature review of the English language literature was conducted using PubMed, MEDLINE, and Embase as per the Preferred Reporting Items for Systematic Reviews and Meta-Analyses (PRISMA) guidelines. Outcomes of interest included 90-day modified Rankin scale (mRS) 0-2, puncture to recanalization time, and thrombolysis in cerebral infarction (TICI) scores 2b/3 and 3. We calculated pooled event rates and their corresponding 95% confidence intervals (CI) for all outcomes.

We included seven studies with 68 patients in our analysis. All patients underwent mechanical thrombectomy via transradial artery access for AIS due to PC-LVO. The pooled meantime of puncture to recanalization was 29.19 (95% CI=24.05 to 35.42) minutes. Successful recanalization (TICI2b/3) was achieved in 98.69% (95% CI=93.50 to 100) of patients and complete recanalization (TICI 3) in 52.16% (95% CI=34.18 to 79.60) of the patients. Overall, 56.84% (95% CI=41.26 to 78.30) of patients achieved mRS 0-2.

Transradial artery access for mechanical thrombectomy for PC-LVO stroke displays early promise and feasibility, particularly regarding very high rates of successful recanalization and low puncture to recanalization time.

## Introduction and background

Acute ischemic stroke (AIS) is a serious clinical event with grim outcomes and significant morbidity and mortality. It is often categorized by the location of the occlusion, typically either as anterior circulation large vessel occlusion (AC-LVO) or posterior circulation large vessel occlusion (PC-LVO) [1]. The latter is defined as an occlusion of the basilar artery or vertebral arteries. A PC-LVO stroke accounts for roughly 25% of all AIS and often presents with different symptoms and etiologies than AC-LVO [2-4].

Mechanical thrombectomy for AIS is most often performed via transfemoral artery access. However, transradial artery access has been proven to be safe and efficacious for AC-LVO [5]. The utilization of the radial artery for neuro-interventional procedures has gained traction following its use as the first-line access route for interventional cardiovascular procedures [6]. Furthermore, current literature suggests that transradial artery access is preferred over transfemoral artery access by patients, who often view transradial access as less stressful, embarrassing, and painful than transfemoral access [7-9]. In addition to patient preference, transradial artery access is often preferred by proceduralists in the setting of different patient anatomies and access site difficulties [5].

While present literature has demonstrated the non-inferiority of transradial artery access for mechanical thrombectomy in AC-LVO, there has, to our knowledge, been no such analysis completed on PC-LVO patients [5]. The objective of this study is to analyze transradial artery access as an option for mechanical thrombectomy in PC-LVO stroke. In this paper, we performed a systematic review and meta-analysis of studies that have reported outcomes for patients undergoing mechanical thrombectomy for PC-LVO via transradial artery access.

## Review

Methods

Literature Search Details

We performed this systematic review and meta-analysis according to the Preferred Reporting Items for Systematic Review and Meta-analyses statement (PRISMA) recommendations [10]. We formulated the patient/population, intervention, comparison, and outcomes (PICO) question according to the following data population: patients with posterior circulation stroke; intervention: transradial mechanical thrombectomy, no specific comparator was proposed; outcomes: puncture to recanalization time, TICI scores, and the associated rates of functional independence. 

After piloting different combinations to collect the appropriate keywords, we developed a comprehensive search term (see Appendices). The search was conducted on 30th March 2022 through the following databases: PubMed, Scopus, Web of Science, and Embase. We did not pose any restrictions on language, sample size, country of origin, or population characteristics in the published literature. We excluded non-original studies (reviews, systematic reviews, and meta-analyses), letters, commentaries, conference abstracts/posters, case reports, and case series with less than five patients. The search was followed by a manual search of reference lists of the included studies. Finally, study metadata and abstracts were uploaded to the AutoLit platform (Nested Knowledge, St. Paul, MN, USA) for screening and extraction.

Screening and Study Selection

The screening was performed using the AutoLit software platform [11] (with previously published methods [12] ). All studies that describe endovascular therapy (EVT) done through transradial access for patients with posterior circulation strokes were screened for relevant information based on our inclusion criteria. The first screening stage was done by checking the title and abstract and was completed by two reviewers, with a third senior author adjudicating all conflicting decisions. This was followed by the second stage of full-text screening of the retained articles to verify the suitability of data extraction and confirm relevance.

Data Extraction

Two independent authors executed the data extraction, and the senior author validated the data to resolve any disagreements through discussion. The extraction sheet included the studies' main characteristics, population demographics, and different outcomes.

Quality Assessment

The Newcastle-Ottawa scale was used to assess the risk of bias, with two independent reviewers evaluating all studies [13]. A third senior reviewer assessed any studies or domains with discrepancies. The Newcastle-Ottawa scale contains eight items within three domains (selection, comparability, and outcome), with a maximum total score of nine. The quality of the studies was given a rating based on the overall score, with more than/equal seven points considered as "good", two to six points considered as "fair", and less than/equal one point considered as "poor" quality.

Statistical Analysis

We pooled prevalence rates and means with the corresponding 95% confidence intervals (95% CI). Random- or fixed-effect models were used to pool the data based on the heterogeneity level among the included studies. Heterogeneity was assessed using Q statistics and the I2 test, where P-value < 0.05 or I2 > 50% were considered significant [14]. The pooled studies were less than ten, so publication bias could not be tested using Egger's regression test [15,16]. All data were analyzed using R software version 4.2.0. and the "meta" package.

Results

Search and Screening Results

**Figure 1:**
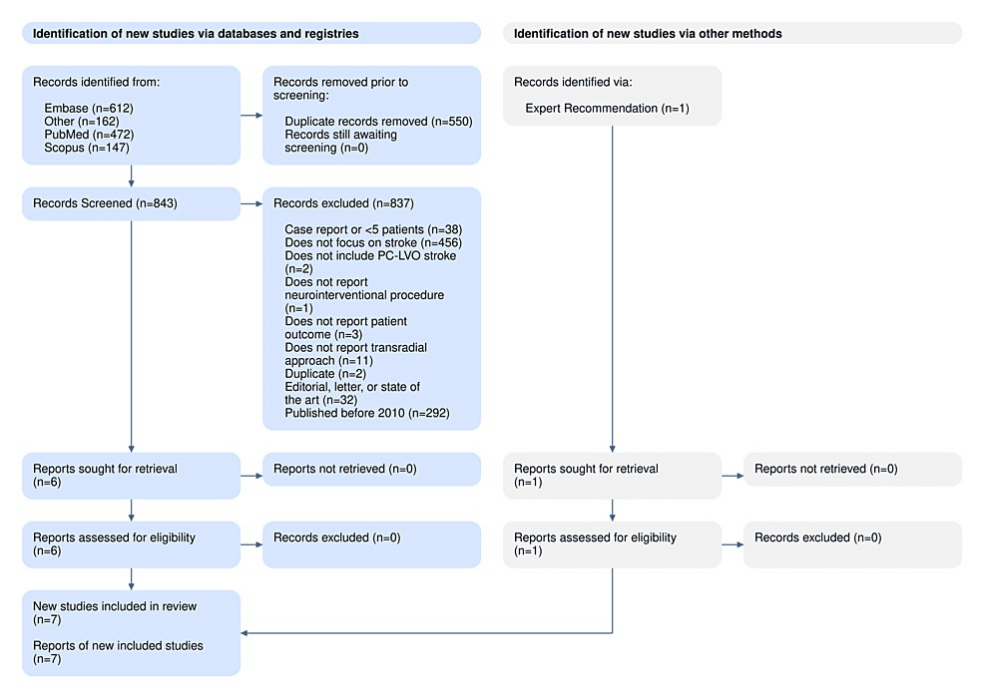
PRISMA flow diagram showing the review process PRISMA: Preferred Reporting Items for Systematic Reviews and Meta-Analyses

We retrieved 1,393 initial records from the queried databases with 550 duplicates. Of the unique 843 studies, 837 articles were excluded, and six articles were sent forward for full-text screening. These six were finally included, along with one study retrieved from a manual search, for a total of seven studies included in the quantitative and qualitative syntheses (Figure 1).

Study Characteristics and Quality Assessment

Of the seven included studies, six used a retrospective design and one was a multicenter study. The sample size of the included studies ranged from two individuals to 23 with reported outcomes at three months of follow-up for all studies. The characteristics of the included studies and patient outcomes are detailed in Table 1.

**Table 1:** Study characteristics and patient outcomes of the studies included in the meta-analysis mRS: Modified Rankin scale, TICI: Thrombolysis in cerebral infarction, SD: Standard of deviation

Study	Sample size	Setting	Puncture to recanalization, minutes, mean (SD)	mRS 0-2, (%)	TICI 2b/3, (%)	TICI 3 (%)
Oselkin et al. 2018 [17]	9	Multi-center	35.8 (25)		88.9	33.3
Crockett et al. 2020 [18]	23	Single-center	27.3 (17.4)	61.1	100	
Maud et al. 2019 [19]	10	Single-center	35.8 (25)		80	60
Pons et al. 2020 [20]	4	Single-center			100	
Scoco et al. 2020 [21]	2	Single-center			100	50
Kuhn et al. 2021 [22]	9	Single-center			100	
Siddiqui et al. 2021 [23]	11	Single-center		45.4		

For all included studies, there was no high risk of bias among all assessed domains. Six of the included studies received a “fair” quality score, and one of the included studies received a “good” quality score. Certain specific bias risk was identified in specific study aspects. A detailed assessment of the risk of bias is represented in Table 2.

**Table 2:** Risk of bias assessment * indicates that the referenced study met the criteria

Newcastle-Ottawa scale assessment (NOS)												
Selection								Comparability	Outcome			
No.	Study	Year	Sample (n)	Representativeness of the exposed cohort	Selection of the non-exposed cohort	Ascertainment of exposure	Demonstration that outcome of interest was not present at start of study	Comparability of cohorts based on the design or analysis	Assessment of outcome	Was follow-up long enough for outcomes to occur?	Adequacy of follow-up of cohorts	Quality Score
1	Oselkin et al. [17]	2018	9	*	N/A	*		*	*	*	*	Fair
2	Crockett et al. [18]	2020	23	*	N/A	*	*	*	*	*	*	Good
3	Satti et al. [19]	2017	10	*	N/A	*		*	*	*	*	Fair
4	Pons et al. [20]	2020	4		N/A	*		*	*	*	*	Fair
5	Scoco et al. [21]	2020	2		N/A	*		*	*	*	*	Fair
6	Kuhn et al. [22]	2021	9	*	N/A	*		*	*	*	*	Fair
7	Siddiqui et al. [23]	2021	11	*	N/A	*		*	*	*	*	Fair

Outcomes

**Figure 2:**
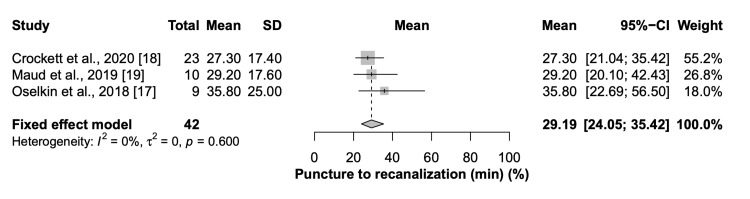
Forest plot of time from puncture to recanalization CI: Confidence interval, SD: Standard of deviation, Min: Minutes

Seven studies with 68 patients were included in the quantitative synthesis with the variability of the outcomes reported within each study. The pooled meantime of puncture to recanalization was 29.19 (95% CI=24.05 to 35.42) minutes (Figure 2).

**Figure 3:**
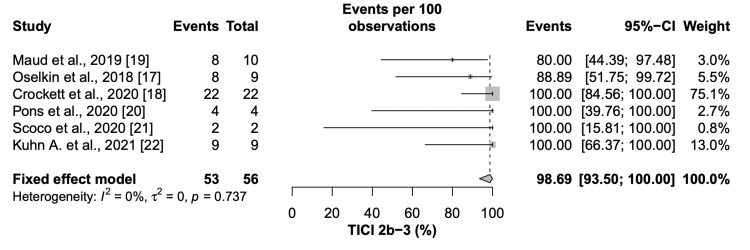
Forest plot of successful recanalization (TICI 2b/3) CI: Confidence interval, TICI: Thrombolysis in cerebral infarction

**Figure 4:**
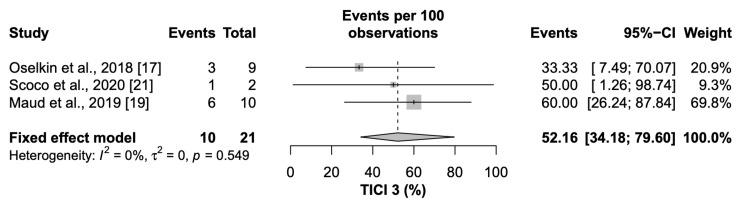
Forest plot of complete recanalization (TICI 3) CI: Confidence interval, TICI: Thrombolysis in cerebral infarction

Successful recanalization (TICI2b/3) and complete recanalization (TICI 3) was achieved in 98.69% (95% CI=93.50 to 100) and 52.16% (95% CI= 34.18 to 79.60) of the patients, respectively (Figure 3, Figure 4).

**Figure 5:**
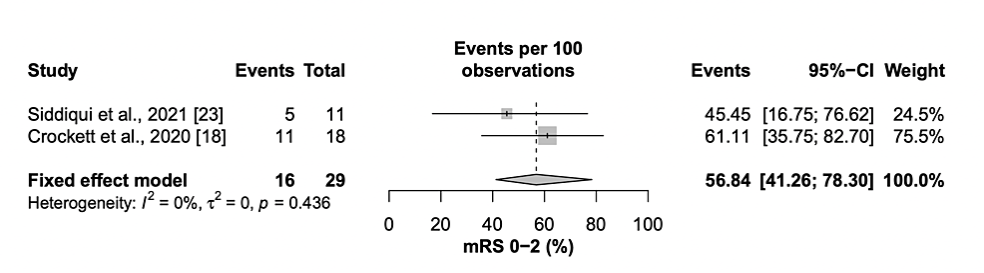
Forest plot of modified Rankin Scale score (mRS) of 0-2 at 90 days

Two studies consisting of a total of 29 patients reported mRS rates with 56.84% (95% CI=41.26 to 78.30) of patients achieving a good functional outcome (mRS 0-2) at 90 days (Figure 5).

There was no heterogeneity among the included studies in all outcomes (I2= 0%; P-value> 0.05).

Discussion

In this meta-analysis and systematic review, we examined the feasibility of the transradial artery as an access site for mechanical thrombectomy for PC-LVO stroke. Our analysis revealed that current literature reports a nearly 100% rate of successful recanalization, short puncture to recanalization times, and good rates of full reperfusion and good functional outcome. These findings are important as they support future investigations into transradial artery access for mechanical thrombectomy for PC-LVO strokes. This is consistent with the findings that have been reported for transradial access in AC-LVO [24]. Our results are also consistent with the Standards and Guidelines Committee of the Society of NeuroInterventional Surgery’s recommendations for transradial artery access as a feasible option for mechanical thrombectomy, particularly in PC-LVO strokes [25].

We found that the mean puncture to recanalization time in our meta-analysis (29.2 minutes) was lower than previous literature had reported for both AC-LVO and PC-LVO strokes performed with transfemoral access and AC-LVO strokes performed with transradial access [24,26-28]. Additionally, it is likely that with increased operator experience, the puncture to recanalization time would decrease further. It has been documented that transradial access requires more technical skill than transfemoral access, meaning that operators would likely benefit from further training and practice [29,30]. Currently, neuro-interventionalists are much more experienced in transfemoral access, suggesting that there is room for improvement with more practice in using the transradial artery as an access site [18,28]. Despite the lower level of experience with transradial access, the studies included in our present analysis showed a promising rate of attainment of transradial approach access.

Previous studies, including the landmark thrombectomy randomized controlled trials, have reported successful recanalization rates between 58% to 88% [31-35]. These findings have historically excluded PC-LVO stroke, focusing only on AC-LVO stroke. Additionally, mechanical thrombectomy in these studies was performed almost exclusively through transfemoral access. Our study reports rates of successful recanalization of 98.7%. It is difficult to compare these results to previous literature. Firstly, as previously mentioned, most published studies did not focus on the same intervention or location of occlusion as our current study. Secondly, our meta-analysis of successful recanalization had a sample size of 56 patients, so any single patient’s results could have a large impact on the overall results of our meta-analysis. Though this sample size is low, it is, to our knowledge, the largest analysis performed on patients with PC-LVO stroke treated with mechanical thrombectomy via transradial artery access. So, despite being underpowered, our results demonstrate that transradial access for PC-LVO stroke treated with mechanical thrombectomy shows early promise and warrants further investigation.

Our present study suffered from multiple limitations. The most glaring of these limitations was the lack of available literature on our topic of interest. As a result, our study was underpowered. This is to be expected as transradial artery access has been underutilized historically, resulting in a limited sample size. Additionally, the majority of strokes occur in the anterior circulation, so available data on posterior circulation stroke as a whole is more limited. Posterior circulation stroke treatment protocol is often different than anterior circulation stroke treatment protocol which may introduce bias to our results. Similarly, operator experience may have played a role in the results of our study as most clinicians have less experience using the transradial artery as an access site. However, despite the low sample size of our analysis, results were consistent throughout most of the studies included in our meta-analysis. Another limitation of our study was the quality of the papers included. Of the seven studies included in our meta-analysis, only one was prospective. Furthermore, multiple studies would have otherwise been valuable for our study but did not stratify outcomes by the location of the occlusion. The papers included were limited in the data supplied, with little information on important prognostic information such as last known normal time, whether transradial access was first-line or bail-out treatment, and patient perfusion study data. Finally, the evidence supporting the TICI score as valid for PC-LVO stroke is lacking.

Our study, while important, demonstrates the need for a larger sample size so that a higher-powered analysis can be performed. At present, results from existing literature are promising and warrant further investigation of transradial artery access for mechanical thrombectomy for PC-LVO stroke. As further higher quality data is collected, clinicians will become better informed on the ideal access site for mechanical thrombectomy in PC-LVO strokes.

## Conclusions

In this meta-analysis regarding the feasibility of transradial access for mechanical thrombectomy in PC-LVO stroke, we found that transradial access is associated with excellent rates of successful recanalization, high rates of complete recanalization, good functional outcome, and a quick puncture to recanalization time. Future prospective studies should focus on patient inclusion criteria and operator preference and experience levels so that a higher-powered meta-analysis can be performed. Also, future studies should further explore transradial access versus transfemoral access specifically in the setting of PC-LVO stroke. Presently, transradial artery access for mechanical thrombectomy shows early promise for the treatment of PC-LVO stroke.

## Appendices

The comprehensive search term created to source literature for our study

(TITLE-ABS((("transradial" OR "transradially") AND ("thrombectomy" OR "thrombectomy")) OR (("transradial" OR "transradially") AND ("mechanical" OR "mechanically" OR "mechanicals" OR "mechanics" OR "mechanics" OR "mechanic") AND ("thrombectomy" OR "thrombectomy")) OR (("transradial" OR "transradially") AND ("approach" OR "approach s" OR "approachability" OR "approachable" OR "approache" OR "approached" OR "approaches" OR "approaching" OR "approachs")) OR (("transradial" OR "transradially") AND ("access" OR "accessed" OR "accesses" OR "accessibilities" OR "accessibility" OR "accessible" OR "accessing")) OR (("radial artery" OR ("radial" AND "artery") OR "radial artery" OR "radial" OR "radially" OR "radials") AND ("access" OR "accessed" OR "accesses" OR "accessibilities" OR "accessibility" OR "accessible" OR "accessing")) OR (("radial artery" OR ("radial" AND "artery") OR "radial artery" OR "radial" OR "radially" OR "radials") AND ("approach" OR "approach s" OR "approachability" OR "approachable" OR "approache" OR "approached" OR "approaches" OR "approaching" OR "approachs")) OR ((("transfemoral" OR "transfemorally") AND ("thrombectomy" OR "thrombectomy")) OR (("transfemoral" OR "transfemorally") AND ("mechanical" OR "mechanically" OR "mechanicals" OR "mechanics" OR "mechanics" OR "mechanic") AND ("thrombectomy" OR "thrombectomy")) OR (("transfemoral" OR "transfemorally") AND ("approach" OR "approach s" OR "approachability" OR "approachable" OR "approache" OR "approached" OR "approaches" OR "approaching" OR "approachs")) OR (("transfemoral" OR "transfemorally") AND ("access" OR "accessed" OR "accesses" OR "accessibilities" OR "accessibility" OR "accessible" OR "accessing")) OR (("femor" OR "femorals" OR "femur" OR "femur" OR "femoral") AND ("access" OR "accessed" OR "accesses" OR "accessibilities" OR "accessibility" OR "accessible" OR "accessing")) OR (("femor" OR "femorals" OR "femur" OR "femur" OR "femoral") AND ("approach" OR "approach s" OR "approachability" OR "approachable" OR "approache" OR "approached" OR "approaches" OR "approaching" OR "approachs"))))) AND (TITLE("posterior stroke" OR "posterior circulation stroke" OR "posterior circulation infarction" OR "posterior circulation infarct" OR "cerebellar infarction" OR "cerebellar stroke" OR "anterior inferior cerebellar artery" OR "posterior inferior cerebellar artery" OR "vertebral" OR "posterior cerebral" OR "superior cerebellar" OR "basilar"))
